# PP106 Links Between Accuracy And Effectiveness Of Laboratory Diagnostic Tests: Health Technology Assessment Of Two Analytical Approaches For Glycated Hemoglobin

**DOI:** 10.1017/S0266462324002678

**Published:** 2025-01-07

**Authors:** Chiara Di Resta, Chiara Sacco, Mladen Trbos, Massimo Locatelli, Rossella Tomaiuolo, Giuseppe Banfi

## Abstract

**Introduction:**

In the field of laboratory medicine, the evolution of knowledge and the innovation of technologies are the basis of analytic and diagnostic progress, leading to the development of new solutions based on innovative technologies. However, these advances must be accompanied by evidence of appropriateness, diagnostic effectiveness, and efficiency of organizational aspects, considering the impact of the test on patient outcomes.

**Methods:**

This study is an exemplificative case of the application of health technology assessment (HTA), exploiting the EUnetHTA core model, on two analyzers able to determine the glycated hemoglobin (hemoglobin A1c, HbA1c), the Capillarys 2 Flex Piercing analyzer (by Sebia, Lisses, France), and the HLC-723G11 analyzer (by Tosoh Corporation, Shunan, Yamaguchi, Japan) in the Laboratory Medicine Service of the IRCCS San Raffaele Hospital (Milan, Italy).

**Results:**

The nine domains of the EUnetHTA model are assessed, comparing the two technologies of interest and highlighting their pros and cons. As described below, the main aspect to be considered is the impact of the testing results on the clinical effectiveness evidence, due to the two different detection methods while both analyzers respond equally to the clinical and organizational needs of the San Raffaele laboratory.

**Conclusions:**

This study demonstrates how HTA can aid decision-makers in evaluating health technologies to achieve specific objectives of diagnosis, treatment, and prevention.

